# Speedy and Spontaneous Recovery from Rupture of Rectum and Bladder

**Published:** 1870-12

**Authors:** O. C. Gibbs

**Affiliations:** Frewsburg, N. Y.


BUFFALO

Medical and Surgical Journal.

VOL. X. DECEMBER, 1870. No. 5.

### Original Communications.

ART. I.—*Speedy and Spontaneous Recovery from Rupture of Rectum and Bladder. By* O. C. GIBBS, M, D., *Frewsburg, N. Y.*

In the summer of 1869, I was called to see Mr. L——, a Swede, aged about 55 years. Being called about bedtime, and the patient living about eight miles away, and the intervening road being quite bad, I did not visit him until the next morning. I found the patient in bed in a log hut, with but one room, and he all alone. The floor was literally covered with blood, and the bed saturated with the same fluid.

On attempting conversation, I found my patient could not speak, or even understand a word of English. An interpreter came to my aid in a few moments. I ascertained that, on the afternoon before, while pitching hay off a wagon, and that the last of the load, and pitching up to a considerable height, his foot slipped and he fell backwards on to a sharpened stake of the rack. The stake entered the anus so centrally as to show very little signs of injury, but passing up must, from the nature of things, have severely lacerated the rectum and bladder. Falling still farther, the stake was broken off, and subsequently withdrawn by his co-workman.

Hemorrhage had nearly ceased, yet I considered it prudent to give him a cold water injection; and, as he had whiskey in the house, I ordered a free dose. Smelling a very strong odor of urine about the house, I enquired if he had passed his urine involuntarily, and learned that, since the injury, he had passed no water by the urethra, but entirely by the anus. No examination per rectum having been made up to this time, I did not know the bladder was injured. As soon as the patient rallied a little, I had him lifted on to his feet, and, while supported, ordered him to attempt to make water while standing. He made the attempt and a stream of urine spurted from the anus.

On laying him down I made repeated attempts to pass a catheter, but his shrieks and contortions from pain compelled me each time to abandon the attempt, and his Swede friends were so alarmed that they insisted upon my abandoning the attempt. Having no chloroform with me, I felt compelled to do so.

The broken stake was shown me: it was of ash, $1\frac{1}{2}$ inches in diameter, and full a foot in length.

Seeing but little I could do for him under the circumstances, I ordered the bed to be changed and floor cleansed; also, cloths put under the hips to catch the urine, which cloths could be removed at pleasure and others substituted, and by no means to let the bed get saturated with urine. I also ordered small doses of opium to be administered every six hours, and an ounce of whiskey every six hours, and such reasonable nourishment as he might desire, and left the case for that day.

Circumstances were such that I could not see him on the succeeding day, but on the second I saw him and found him comfortable, without any very great vascular excitement. He still passed his urine from the anus. He positively refused to have another attempt made to pass the catheter. My design was to pass a gum-elastic catheter and leave it there, through which the urine might pass, and thus avoid its irritating effects upon the wounded surfaces. He also refused to take any medicines.

If my memory serves me right, I prevailed upon him to take wintergreen tea and drink elm water. I now left the case, telling the friends that, as he would submit to no treatment, it was useless to visit him, and I should only come when called.

I heard no more from the case for several weeks, when, on seeing a friend of his and making enquiry, I learned that, within three or four days from my last visit, he began to pass urine slightly by the urethra, and, after a few more days, he had full control of the urine and passed it entire by the urethra. After about two weeks from the date of the injury he was out doing light work, and, after a few weeks more, went to work on a railroad then being constructed, with shovel and barrow, doing full day's work, at which kind of labor he is still engaged.

I should have previously stated that this man was but a few weeks in this country, his wife and family remaining behind. This accounts for his being and living alone—cooking his own food, out of economy, that he might the sooner lay up money enough to enable him to send for family.

This is certainly one of those cases manifesting strong natural recuperative powers. A strong desire and determination to live for his family, and to consummate the object for which he was so diligently laboring had, probably, some influence over the result. Had he been discouraged, homesick, despondent, and given up in despair, he would, quite likely, have died. Mental influences have much to do with the physical recuperative energies.

